# Comparative outcomes and prognostic indicators in adrenalectomy for adrenal metastasis

**DOI:** 10.1007/s00464-024-10691-4

**Published:** 2024-02-05

**Authors:** JungHak Kwak, Hye Lim Bae, Younghoon Jung, Jaebong Choi, Hyeonuk Hwang, Jung Hee Kim, Su-jin Kim, Kyu Eun Lee

**Affiliations:** 1 Department of Surgery, Seoul National University Hospital, Seoul, Korea; 2 Department of Surgery, Seoul National University College of Medicine, Seoul, Korea; 3 Division of Endocrine Surgery, Department of Surgery, Gibbeum Hospital, Seoul, Korea; 4 Department of Internal Medicine, Seoul National University Hospital, Seoul, Korea; 5 Department of Internal Medicine, Seoul National University College of Medicine, Seoul, Korea; 6 Cancer Research Institute, Seoul National University College of Medicine, Seoul, Korea; 7 Medical Big Data Research Center, Institute of Medical and Biological Engineering, Seoul National University, Seoul, Korea

**Keywords:** Adrenalectomy, Laparoscopic surgical procedure, Metastases, Recurrence, Survival

## Abstract

**Purpose:**

The indications for adrenalectomy and feasibility of laparoscopic adrenalectomy for adrenal metastasis are controversial. This study aimed to compare the surgical outcomes between open adrenalectomy (OA) and laparoscopic adrenalectomy (LA) and to evaluate the prognostic factors for oncological outcomes of adrenal metastasis.

**Materials and Methods:**

We conducted a retrospective chart review of 141 consecutive patients who underwent adrenalectomy for adrenal metastasis at Seoul National University Hospital from April 2005 to February 2021. Surgical and oncological outcomes were compared between OA and LA.

**Results:**

OA was performed in 95 (67.4%) patients, and 46 (32.6%) patients underwent LA. Among the patients who underwent adrenalectomy without adjacent organ resection for adrenal tumors less than 8 cm, LA was associated with a shorter operation time (100.1 ± 48.8 vs. 158.6 ± 81.2, *P* = 0.001), less blood loss (94.8 ± 93.8 vs. 566.8 ± 1156.0, *P* = 0.034), and a shorter hospital stay (3.7 ± 1.3 vs. 6.9 ± 5.8, *P* = 0.003). For locoregional recurrence-free survival (LRRFS), on multivariate analysis, a positive pathological margin (hazard ratio [HR]: 5.777, *P* = 0.002), disease activity at the primary site (HR: 6.497, *P* = 0.005), other metastases (HR: 4.154, *P* = 0.015), and a relatively larger tumor size (HR: 1.198, *P* = 0.018) were significantly associated with poor LRRFS. Multivariate analysis indicated that metachronous metastasis (HR: 0.51, *P* = 0.032) was associated with a longer overall survival (OS), whereas a positive pathological margin (HR: 2.40, *P* = 0.017), metastases to other organs (HR: 2.08, *P* = 0.025), and a relatively larger tumor size (HR: 1.11, *P* = 0.046) were associated with a shorter OS.

**Conclusions:**

LA is a feasible treatment option for adrenal metastasis in selected patients. The pathological margin, metastases to other organs, and tumor size should be considered in adrenalectomy for adrenal metastasis.

**Supplementary Information:**

The online version contains supplementary material available at 10.1007/s00464-024-10691-4.

The adrenal gland is a common site for metastasis of malignancies from the lung, kidney, liver, breast, colon, and other primary sites [1]. The prevalence of adrenal metastases has been reported to be approximately 3.1% to 33% [2]. Patients with metastatic cancer to other organs are mostly considered to have stage IV disease, which needs palliative or supportive treatment. However, with improvements in systemic therapy, including chemotherapy and immunotherapy, several studies have demonstrated increased survival benefits of adrenalectomy compared with nonsurgical treatment alone for metastatic adrenal disease [3].

Depending on the origin of the primary cancer, heterogeneous long-term survival outcomes after adrenalectomy for adrenal metastasis have been reported. Krumeich et al. reported that lung cancer origin showed a durable survival outcome in median disease-free survival (DFS) (40 months; 1 year 64.8%, 5 year 42.9%) and overall survival (OS) (47 months; 1 year 80.2%, 5 year 35.2%) after adrenalectomy for metastatic adrenal disease [4]. Vlk et al. reported that for patients who underwent adrenalectomy for metastatic adrenal disease, renal and colon cancer patients had longer median survival times (37 months (IQR 13–82) and 35 months (IQR 12–83), respectively) than lung cancer patients (16 months (IQR 8–48)) [5].

According to current guidelines, adrenalectomy for metastatic adrenal disease can prolong DFS and OS in selected patient groups [1, 6]. Several factors, including the status of primary cancer, the number of metastatic sites, and the time interval between primary cancer to adrenal metastasis, need to be considered when selecting patients for this treatment [7]. Nevertheless, there are no definitive surgical indications for adrenalectomy for metastasis or prognostic factors related to survival outcomes.

With the development and advancement of minimally invasive surgery, the laparoscopic approach for adrenalectomy has been performed as the treatment of choice for metastatic disease [8]. However, if the adrenal tumor has malignant features, such as a large size, and is suspected to invade adjacent organs, conventional open surgery should be considered [9]. There are concerns about incomplete resection or tumor rupture, which may occur during the laparoscopic approach and could lead to the locoregional recurrence of metastatic cancer. Therefore, the indications for laparoscopic surgery for adrenal metastasis are unclear.

This study aimed to evaluate the prognostic factors of locoregional recurrence-free survival (LRRFS) and OS after adrenalectomy for adrenal metastasis and to compare the surgical outcomes between laparoscopic and conventional open adrenalectomy for adrenal metastasis.

## Materials and methods

### Patients

From April 2005 to February 2021, a retrospective chart review was performed of patients who underwent adrenalectomy for metastatic adrenal disease at Seoul National University Hospital. A total of 141 consecutive patients were identified. Patient clinicopathological data, operative approach, complications, locoregional recurrence, and survival data were collected. This study was approved by the Institutional Review Board of Seoul National University Hospital (IRB number: 2204-077-1316).

### Definitions

The timing of adrenal metastasis from the primary tumor was defined as the period from the diagnosis date of primary cancer to the diagnosis date of adrenal metastasis; synchronous metastasis was defined as the detection of adrenal metastasis within 6 months after diagnosis of the primary tumor, and metachronous metastasis was defined as the detection of adrenal metastasis 6 months after diagnosis of the primary tumor. Locoregional recurrence was defined as radiological confirmation of recurrence in the previous adrenalectomy site. LRRFS was measured from the date of adrenalectomy to the date of the diagnosis of locoregional recurrence or the last follow-up. OS was defined as the interval from the date of adrenalectomy to the date of the last follow-up or death. Adjuvant systemic or radiation therapy was defined as any systemic or radiation treatment undergone after adrenalectomy.

### Surgical intervention

Open, laparoscopic (or retroperitoneoscopic), and robotic adrenalectomies were performed by transperitoneal or retroperitoneoscopic approaches. To compare the conventional open approach and laparoscopic approach, patients were grouped based on the type of surgical approach; open and conversion to open surgeries were defined as the open approach group, and laparoscopic, retroperitoneoscopic and robotic surgeries were defined as the laparoscopic approach group. Surgeons chose the operative approach based on each patient’s clinical characteristics. Conventional open adrenalectomy was performed in patients with a relatively large tumor size, suspected invasion to adjacent organs or previous abdominal operation histories.

### Statistical analysis

Continuous variables are presented as the mean with standard deviation or the median with interquartile range (IQR) as appropriate, and these data were analyzed by Mann–Whitney *U* tests. Categorical variables were analyzed using a chi-square test to compare the clinicopathological variables. Survival rates were calculated using Kaplan‒Meier survival analysis, with differences analyzed with the log-rank test. Univariate and multivariate Cox regression analyses were used to evaluate clinical characteristics associated with LRRFS and OS. *P* < 0.05 was defined as statistically significant. Statistical analyses were performed using R statistical software version 4.2.2 (http://www.R-project.org).

## Results

### Patient characteristics

During the study period, 141 patients underwent adrenalectomy for metastatic adrenal disease (Table 1). The most common type of cancer site was the kidney (39, 27.7%), followed by the liver (33, 23.4%), lung (24, 17.0%), colon (16, 11.3%), and others (29, 20.6%). The other primary cancers were sarcoma, breast cancer, stomach cancer, melanoma, etc. (Table S1). In patients with bilateral adrenal metastasis, synchronous contralateral adrenal metastasis was detected in 18 (51.4%) after the initial diagnosis of adrenal metastasis. Locoregional recurrence was detected in 38 (27.0%) patients, and the median locoregional recurrence-free interval was 18.0 (IQR 8.0–50.0) months. Unfortunately, 98 (69.5%) patients died during the study period. The median survival time after adrenalectomy was 25.0 (IQR 13.0–54.0) months.

**Table 1:** Patient characteristics

Variables	N (= 141) (%)
Age (years), mean ± SD	60.3 ± 11.3
Sex	
Male	105 (74.5%)
Female	36 (25.5%)
BMI (kg/m^2^), mean ± SD	23.9 ± 3.3
Primary cancer	
Kidney	39 (27.7%)
Liver	33 (23.4%)
Lung	24 (17.0%)
Colon	16 (11.3%)
Others	29 (20.6%)
Timing of adrenal metastasis from a primary tumor	
Synchronous (≤ 6 mo)	50 (35.5%)
Metachronous (> 6 mo)	89 (63.1%)
Unknown	2 (1.4%)
Mean time from primary to metastasis (months), median (IQR)	17.0 (1.0–40.0)
Location of the adrenal metastasis	
Unilateral	106 (75.2%)
Bilateral	35 (24.8%)
Synchronous (≤ 6 mo)	18 (51.4%)
Metachronous (> 6 mo)	17 (48.6%)
Mean tumor size of adrenal metastasis (cm), mean ± SD	3.7 ± 2.5
Locoregional recurrence	38 (27.0%)
Death	98 (69.5%)
Locoregional recurrence free interval (months), median (IQR)	18.0 (8.0–50.0)
Survival time after adrenalectomy (months), median (IQR)	25.0 (13.0–54.0)
Follow-up duration (months), median (IQR)	23.0 (10.5–50.0)

*SD* standard deviation, *BMI* body mass index, *IQR* interquartile range

### Treatment of primary cancer and adrenal metastasis

The details of treatment for primary cancers and adrenal metastasis are shown in Table S2. Among 141 patients, open adrenalectomy was performed in 88 (62.4%) patients, and seven (4.7%) patients had a conversion to open adrenalectomy. Laparoscopic (or retroperitoneoscopic) adrenalectomy was performed in 42 (29.8%) patients; 36 patients underwent a laparoscopic approach, and 6 patients underwent a retroperitoneoscopic approach. Four (2.8%) patients underwent robotic adrenalectomy.

### Comparison of surgical outcomes between open and laparoscopic adrenalectomy

A comparison of operative details between open and laparoscopic approaches is shown in Table 2. In terms of the surgical extent of adrenalectomy, a significant difference was found between the two groups (*P* < 0.001), namely, more patients in the open approach group had multiple organ resection. Compared to the laparoscopic approach, adrenalectomy with the open approach was associated with a significantly longer operation time (212.6 ± 113.5 min vs. 112.0 ± 63.5 min, *P* = 0.001), greater estimated blood loss (869.6 ± 1494.4 ml vs. 118.3 ± 123.0 ml, *P* = 0.001), longer postoperative hospital stay (8.9 ± 6.4 days vs. 4.3 ± 2.8 days, *P* = 0.001), and higher rates of postoperative complication rates (52.6% vs. 19.6%, *P* = 0.001). Locoregional recurrence was not different between the open and laparoscopic approaches (27 (29.0%) vs. 11 (23.9%), *P* = 0.524). Death was not different between the open and laparoscopic approaches (70 (73.7%) vs. 28 (60.9%), *P* = 0.121).

**Table 2:** Comparison of surgical outcomes between open and laparoscopic adrenalectomy

	Open approach (n = 95)	Laparoscopic approach (n = 46)	P value
Extent of adrenalectomy			< 0.001
Adrenalectomy	45 (47.4%)	42 (91.3%)	
Adrenalectomy with single organ resection	32 (33.7%)	1 (2.2%)	
Adrenalectomy with multiple organ resection	18 (18.9%)	3 (6.5%)	
Pathological margin			0.122
Negative	49 (81.7%)	34 (94.4%)	
Positive	11 (18.3%)	2 (5.6%)	
Operation time (min)	212.6 ± 113.5	112.0 ± 63.5	< 0.001
Tumor size (cm)	4.0 ± 2.9	3.1 ± 1.8	0.052
Operative blood loss (ml)	869.6 ± 1494.4	118.3 ± 123.0	< 0.001
Length of hospital stay (days)	8.9 ± 6.4	4.3 ± 2.8	< 0.001
Intraoperative complications	15/95 (15.8%)	2/46 (4.3%)	0.055
None	80 (84.2%)	44 (95.7%)	
Vascular injury	3 (3.2%)	1 (2.3%)	
Bowel injury	5 (5.4%)	1 (2.3%)	
Diaphragm injury	4 (4.3%)	0 (0%)	
Others	3 (3.2%)	0 (0%)	
Postoperative complications	50/95 (52.6%)	9/46 (19.6%)	< 0.001
None	45 (47.4%)	36 (78.3%)	
Ileus	13 (13.7%)	3 (6.5%)	
Wound problems	6 (6.3%)	0 (0.0%)	
Atelectasis	31 (32.6%)	5 (10.9%)	
Pneumonia	4 (4.2%)	1 (2.2%)	
ACS	1 (1.1%)	0 (0.0%)	
Others	6 (6.3%)	1 (2.2%)	
Locoregional recurrence	27 (29.0%)	11 (23.9%)	0.524
Death	70 (73.7%)	28 (60.9%)	0.121
Follow up duration (months), median (IQR)	22.0 (10.0—50.0)	23.5 (13.3 – 52.5)	0.542

*IQR* interquartile range

### Comparison of surgical outcomes between open and laparoscopic adrenalectomy without adjacent organ resection for adrenal tumors smaller than 8 cm

To determine the surgical and oncological outcomes of laparoscopic adrenalectomy compared to the open approach, we performed subgroup analysis among the patients who did not undergo combined resection with other organs for adrenal tumors less than 8 cm (n = 73) (Table 3). Compared to open adrenalectomy (n = 34), laparoscopic adrenalectomy (n = 39) was associated with a significantly shorter operation time (158.6 ± 81.2 min vs. 100.1 ± 48.8 min, *P* = 0.001), less estimated blood loss (3.5 ± 1.7 cm vs. 2.8 ± 1.3 cm, *P* = 0.034), and a shorter postoperative hospital stay (6.9 ± 5.8 days vs. 3.7 ± 1.3 days, *P* = 0.003). There was no significant difference in the pathological margin, tumor size, overall postoperative complications, locoregional recurrence, or death between the groups.

**Table 3:** Comparison of surgical outcomes between open and laparoscopic adrenalectomy without adjacent organ resection for adrenal tumor less than 8 cm

	Open approach (n = 34)	Laparoscopic approach (n = 39)	P value
Pathologic margin			0.215
Negative	19 (82.6%)	31 (93.9%)	
Positive	4 (17.4%)	2 (6.1%)	
Operation time (min)	158.6 ± 81.2	100.1 ± 48.8	0.001
Tumor size (cm)	3.5 ± 1.7	2.8 ± 1.3	0.062
Operative blood loss (ml)	566.8 ± 1156.0	94.8 ± 83.8	0.034
Length of hospital stay	6.9 ± 5.8	3.7 ± 1.3	0.003
Intraoperative complication	7/34 (20.6%)	2/39 (5.1%)	0.073
None	27 (79.4%)	37 (94.9%)	
Vascular injury	1 (2.9%)	1 (2.6%)	
Bowel injury	2 (5.8%)	1 (2.6%)	
Diaphragm injury	2 (5.8%)	0 (0.0%)	
Others	2 (5.8%)	0 (0.0%)	
Postoperative complication	13/34 (38.2%)	8/39 (20.5%)	0.095
None	21 (61.8%)	31 (79.5%)	
Ileus	4 (11.8%)	3 (7.7%)	
Wound problems	3 (8.8%)	0 (0.0%)	
Atelectasis	8 (23.5%)	5 (12.8%)	
Pneumonia	0 (0.0%)	0 (0.0%)	
ACS	0 (0.0%)	0 (0.0%)	
Others	1 (2.9%)	1 (2.6%)	
Locoregional recurrence	10 (29.4%)	9 (23.1%)	0.538
Death	26 (76.5%)	22 (56.4%)	0.072
Mean follow-up duration (months), median (IQR)	43.5 (19.0—67.8)	26.0 (15.0–54.0)	0.255

*ACS* acute coronary syndrome, *IQR* interquartile range

### Oncological outcomes: locoregional recurrence

**Fig. 1:**
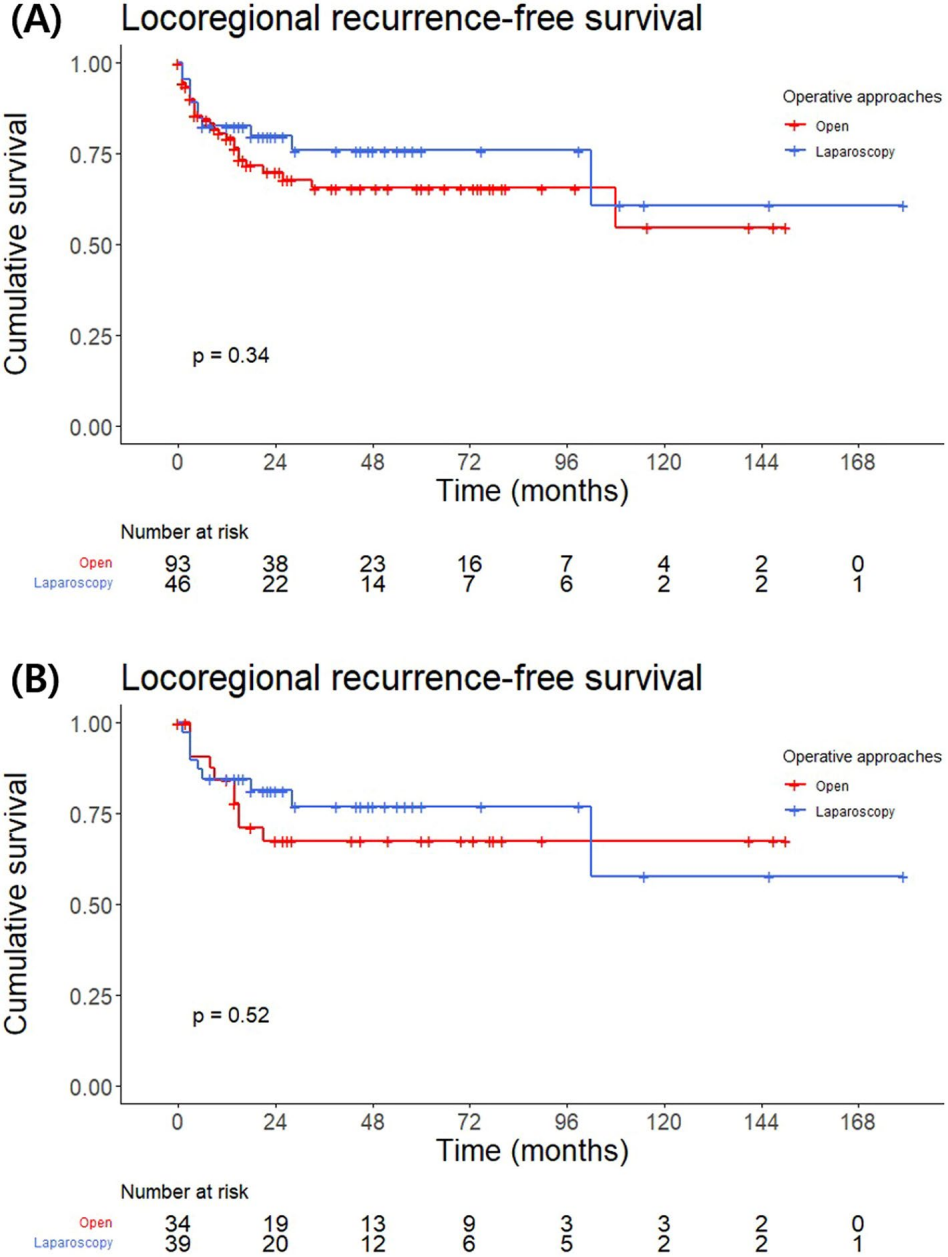
Kaplan–Meier locoregional recurrence-free survival estimates by operative approaches: **A** open and laparoscopic adrenalectomy (p = 0.343), **B** open and laparoscopic adrenalectomy without adjacent organ resection for the tumor smaller than 8 cm (p = 0.521)

After adrenalectomy for adrenal metastasis, the 1-year LRRFS was 80.5%, and the 5-year LRRFS was 69.3% (Fig. S1). A multivariate Cox regression analysis indicated that a positive pathological margin (HR: 5.777, *P* = 0.002), the presence of disease activity at the time of adrenalectomy at the primary site (HR: 6.497, *P* = 0.005), other metastases (HR: 4.154, *P* = 0.015), and a relatively large tumor size (HR: 1.198, *P* = 0.018) were significantly associated with poor LRRFS (Table 4). The LRRFS of patients who underwent open adrenalectomy was not significantly different from that of patients who underwent laparoscopic adrenalectomy (*P* = 0.343) or adrenalectomy without adjacent organ resection for tumors smaller than 8 cm (*P* = 0.521) (Fig. 1).

**Table 4:** Univariate and multivariate analyses for locoregional recurrence-free survival

	Univariable			Multivariable		
	HR	95% CI	P value	HR	95% CI	P value
Age (years)	1.008	0.977–1.039	0.632			
Sex						
Male	Ref	–	–			
Female	0.508	0.212–1.216	0.128			
BMI (kg/m^2^)	0.943	0.856–1.039	0.234			
Operative approach						
Open approach	Ref	–	–			
Laparoscopic approach	0.737	0.365–1.487	0.394			
Primary cancer						
Lung	Ref	–	–			
Renal	0.327	0.095–1.119	0.075			
Liver	1.116	0.423–2.945	0.824			
Colon	1.734	0.601–5.002	0.309			
Others	1.399	0.530–3.696	0.498			
Location of the adrenal metastasis						
Unilateral	Ref	–	–			
Bilateral	1.659	0.856–3.217	0.134			
Extent of adrenalectomy						
Only adrenalectomy	Ref	–	–			
Adrenalectomy with single organ resection	0.736	0.335–1.618	0.446			
Adrenalectomy with multiple organ resection	0.317	0.075–1.335	0.117			
Timing of adrenal metastasis from a primary tumor						
Synchronous (≤ 6 mo)	Ref	–	–			
Metachronous (> 6 mo)	1.043	0.523–2.082	0.904			
Pathologic margin						
Negative	Ref	–	–			
Positive	8.341	3.659–19.016	< 0.001	5.777	1.882–17.734	0.002
Disease activity at time of adrenalectomy						
None	Ref	–	–			
Primary	1.586	0.672–3.744	0.293	6.497	1.757–24.029	0.005
Other metastasis	1.856	0.904–3.814	0.092	4.154	1.326–13.015	0.015
Size of adrenal tumor (cm)	1.261	1.129–1.409	< 0.001	1.198	1.032–1.391	0.018

*95% CI* 95% confidence interval, *HR* hazard ratio, *BMI* body mass index

### Oncological outcomes: overall survival

**Fig. 2:**
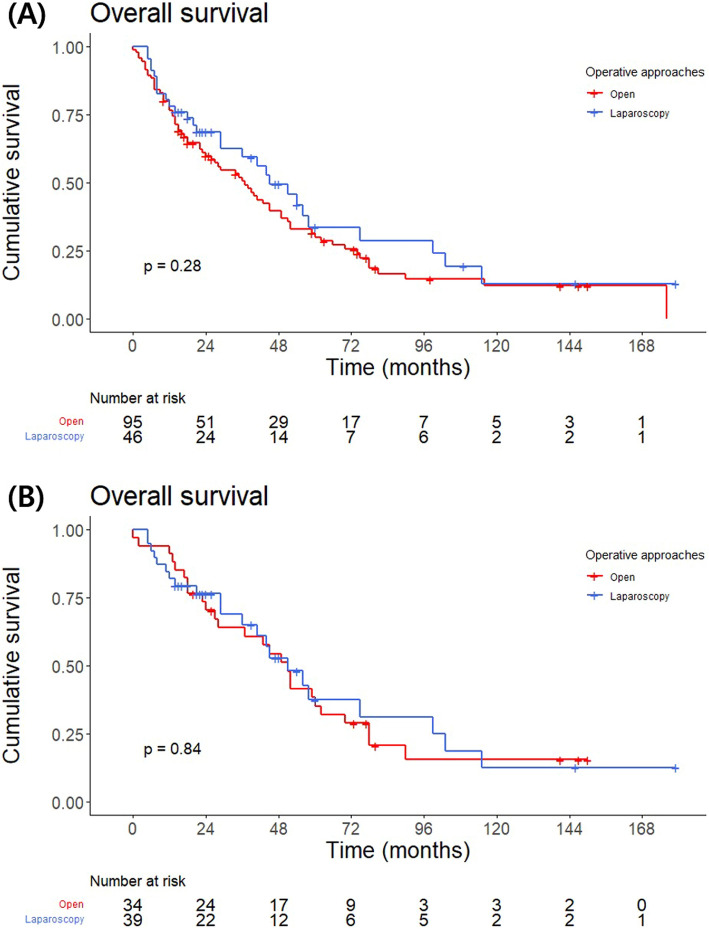
Kaplan–Meier overall survival estimates by operative approaches: **A** open and laparoscopic adrenalectomy (p = 0.278), **B** open and laparoscopic adrenalectomy without adjacent organ resection for the tumor smaller than 8 cm (p = 0.841)

After adrenalectomy for adrenal metastasis, the 1-year OS was 77.2%, and the 5-year OS was 31.4% (Fig. S1). On multivariate analysis, a longer OS was associated with metachronous adrenal metastasis from primary cancer (HR: 0.507, *P* = 0.032). A positive pathological margin (HR: 2.397, *P* = 0.017), presence of metastases to other organs at the time of adrenalectomy (HR: 2.078, *P* = 0.025), and a relatively large adrenal tumor (HR: 1.11, *P* = 0.046) were associated with a shorter OS (Table 5). The OS of patients who underwent open adrenalectomy was not significantly different from that of patients who underwent laparoscopic adrenalectomy (*P* = 0.278) or adrenalectomy without adjacent organ resection was performed for tumors smaller than 8 cm (*P* = 0.841) (Fig. 2).

**Table 5:** Univariate and multivariate analyses for overall survival

	Univariable			Multivariable		
	HR	95% CI	P value	HR	95% CI	P value
Age (years)	0.997	0.977–1.018	0.794			
Sex						
Male	Ref	–	–			
Female	0.972	0.616–1.533	0.903			
BMI (kg/m^2^)	0.930	0.875–0.988	0.019	0.941	0.873–1.015	0.116
Operative approach						
Open approach	Ref	–	–			
Laparoscopic approach	0.786	0.506–1.220	0.283			
Primary cancer						
Lung	Ref	–	–			
Renal	0.884	0.476–1.645	0.698			
Liver	1.179	0.637–2.185	0.600			
Colon	1.031	0.483–2.203	0.937			
Others	1.602	0.850–3.016	0.145			
Location of the adrenal metastasis						
Unilateral	Ref	–	–			
Bilateral	0.977	0.630–1.515	0.917			
Extent of adrenalectomy						
Only adrenalectomy						
Adrenalectomy with single organ resection	1.276	0.804–2.027	0.301			
Adrenalectomy with multiple organ resection	1.223	0.656–2.282	0.526			
Timing of adrenal metastasis from a primary tumor						
Synchronous (≤ 6 mo)	Ref	–	–	Ref	–	–
Metachronous (> 6 mo)	0.574	0.382–0.863	0.008	0.507	0.273–0.943	0.032
Pathologic margin						
Negative	Ref	–	–	Ref	–	–
Positive	2.189	1.164–4.115	0.015	2.397	1.170–4.910	0.017
Disease activity at the time of adrenalectomy						
None						
Primary	1.878	1.123–3.143	0.016	1.758	0.821–3.763	0.146
Other metastasis	2.241	1.402–3.584	0.001	2.078	1.099–3.929	0.025
Size of adrenal tumor (cm)	1.176	1.078–1.283	< 0.001	1.105	1.002–1.220	0.046

*95% CI* 95% confidence interval, *HR* hazard ratio, *BMI* body mass index

## Discussion

Adrenalectomy has been increasingly performed to treat adrenal metastases because resection of adrenal metastases may improve survival benefits [2, 10]. The current international guidelines of the American Association of Clinical Endocrinologists (AACE), American Association of Endocrine Surgeons (AAES) and European Society of Endocrine Surgeons (ESES) for the treatment of adrenal metastases suggest that adrenalectomy for adrenal metastasis is rarely indicated but may improve survival outcomes in a highly selected group of patients [6, 9]. Considerations should be given to pathological results, presentation timing from primary cancers, disease-free interval, and tumor size before adrenalectomy [1, 6]. The clinical outcome benefits can be explained by the recent advancement of minimally invasive surgery, improvement of systemic adjuvant therapy, and appropriate patient selection [8].

In the present study of the clinical outcomes of patients who underwent adrenalectomy for adrenal metastases, we found that laparoscopic adrenalectomy achieved favorable surgical outcomes and no significant difference in locoregional recurrence and death compared to open adrenalectomy. Since more patients in the open adrenalectomy group had multiple organ resection at the time of adrenalectomy, advanced disease requiring adjacent organ resection or reconstruction factors could inform the choice of the open approach rather than the laparoscopic approach. To avoid selection bias, we analyzed patients who underwent adrenalectomy without adjacent organ resection for adrenal tumors smaller than 8 cm and showed consistent clinical outcomes except for the postoperative complication rates.

Previous studies have reported the survival outcome of adrenalectomy for adrenal metastasis based on laparoscopic and open surgical approaches. Moreno et al. found that a patient group who underwent laparoscopic adrenalectomy for adrenal metastasis had a better median (IQR) survival compared to those who underwent open adrenalectomy (45.0 months; IQR 22.6–67.4 vs. 24.0 months; IQR 21.4–26.6, *P* = 0.008), which may be affected by several factors, including smaller tumor sizes, fewer patients who underwent extended adrenalectomy, and higher rates of R0 resection [11]. Goto et al. demonstrated that extra-adrenal metastasis at the time of adrenalectomy (HR 4.13, *P* = 0.022) and a positive surgical margin (HR 2.95, *P* = 0.049) were significantly associated with poor OS after laparoscopic adrenalectomy for adrenal metastasis [12]. Moreno et al. reported that synchronous metastasis was associated with a worse overall survival (HR 1.44, *P* = 0.041) [13]. However, other studies demonstrated no significant difference between synchronous and metachronous metastasis in survival outcomes [14, 15]. In the present study, we found that metachronous metastasis was associated with a better OS (HR 0.51, *P* = 0.032). Patients with metachronous metastasis may have indolent disease since the time to detection of adrenal metastasis from primary cancer in these patients is relatively long.

Although heterogeneous primary cancers may contribute as a bias to the survival outcome in our study cohort, durable survival was also observed after adrenalectomy in patients with adrenal metastasis, which seems to be consistent with previous studies [5, 16]. Based on the present study's findings, several prognostic factors for LRRFS and OS were identified. Positive resection margins, the presence of disease activity at the time of adrenalectomy, and a relatively large tumor size were significantly associated with lower rates of LRRFS and OS. Synchronous metastasis from primary cancer to the adrenal gland was an additional risk factor for poor OS. These findings may provide crucial evidence for deciding the surgical treatment plan for patients with adrenal metastasis of various primary cancers.

To the best of our knowledge, the present study is the first to analyze the clinical outcomes of patients who underwent adrenalectomy alone without adjacent organ resection for adrenal tumors smaller than 8 cm to minimize the selection bias of surgical approaches. Metastatic adrenal disease is often associated with a large tumor size or invasion of adjacent organs, which requires en bloc multivisceral resection via an open approach for a safe pathological margin around structures. This group of patients who underwent open adrenalectomy may have high complication rates and low oncological results. The size limit for the laparoscopic approach for large adrenal tumors has not been established, but recent data have demonstrated that if an adrenal tumor is larger than 8 cm, open adrenalectomy should be performed rather than a laparoscopic approach since it is related to worse surgical outcomes and risk of local invasion to surrounding organs [17–20]. Therefore, we compared the two groups of patients who underwent adrenalectomy alone with adrenal tumors smaller than 8 cm. We analyzed locoregional recurrence between the open and laparoscopic groups of patients. In our study, there were no significant differences in postoperative complications, pathological margins, or locoregional recurrence between the open and laparoscopic approaches.

This study has several limitations. The retrospective design and small number of patients from a single institution influence the results due to selection bias. Various types of primary cancer, which have different disease natures, can introduce heterogeneity in clinical outcomes. Over the long follow-up period, protocols for the management of metastatic adrenal disease were not consistent. The surgical approach regarding open or laparoscopic adrenalectomy was determined by the surgeons based on the patient’s clinical characteristics; tumor size and invasion to adjacent organs factored into the choice of operative approach. However, the present study is sufficient to analyze the surgical outcomes between open and laparoscopic adrenalectomy, including surgical margin, operation time, length of hospital stay, and postoperative complication. A future study of a large number of patients representing a multicenter prospective cohort is important to provide strong evidence.

In conclusion, laparoscopic adrenalectomy is a feasible treatment option for adrenal metastasis in selected patients. When performing adrenalectomy for adrenal metastasis, the pathological margin, disease activity at the time of adrenalectomy, and tumor size should be considered. For the decision regarding adrenalectomy for adrenal metastasis, multidisciplinary and global evaluations of these patients are critical.

### Supplementary Information

Below is the link to the electronic supplementary material.

Supplementary file1 (DOCX 168 KB)
