# Supplementary material for: Comparative outcomes and prognostic indicators in adrenalectomy for adrenal metastasis
Source: Surg Endosc. 2024 Feb 5;38(4):1884–93. doi: 10.1007/s00464-024-10691-4 (PMC10978604; doi:10.1007/s00464-024-10691-4)

**Supplementary table 1. Details of primary cancers**

| **Primary cancer** |  |
| --- | --- |
| Lung (NSCLC) | 24 |
| Renal | 39 |
| Liver | 33 |
| Colon | 16 |
| Sarcoma | 5 |
| Breast | 4 |
| Stomach | 3 |
| Melanoma | 2 |
| Ovary | 2 |
| Bladder | 2 |
| Bile duct | 2 |
| Cervix | 2 |
| Pancreas | 2 |
| Esophagus | 1 |
| Testis | 1 |
| Uterine | 1 |
| MUO | 2 |

**Supplementary table 2. Treatments of primary cancer and adrenal metastasis**

| **Variables** | **N (=141)** |
| --- | --- |
| **Operative approach** |  |
| Open | 88 (62.4%) |
| Laparoscopic | 42 (29.8%) |
| Robotic | 4 (2.8%) |
| Conversion to open | 7 (4.7%) |
| **Disease activity at the time of adrenalectomy** |  |
| None | 67 (47.5%) |
| Primary cancer | 27 (19.1%) |
| Other metastases (± primary ) | 47 (33.3%) |
| **Adjuvant systemic therapy** |  |
| No | 97 (68.8%) |
| Chemotherapy | 42 (29.8%) |
| Immunotherapy | 7 (5.0%) |
| **Adjuvant radiation therapy** |  |
| No | 95 (67.4%) |
| Yes | 46 (32.6%) |
| **Initial treatment (first definitive treatment) of primary tumor** |  |
| None (MUO) | 2 (1.4%) |
| Surgery | 112 (79.4%) |
| Surgery and adjuvant therapy | 15 (10.6%) |
| Adjuvant therapy | 12 (8.5%) |
| **Treatment of contralateral adrenal metastasis** |  |
| No | 10 (28.6%) |
| Adrenalectomy | 5 (14.3%) |
| Chemotherapy | 13 (37.1%) |
| Radiotherapy | 5 (14.3%) |
| Chemotherapy + Radiotherapy | 2 (5.7%) |

**Supplementary figure 1. Kaplan–Meier survival estimates of locoregional recurrence-free survival and overall survival**


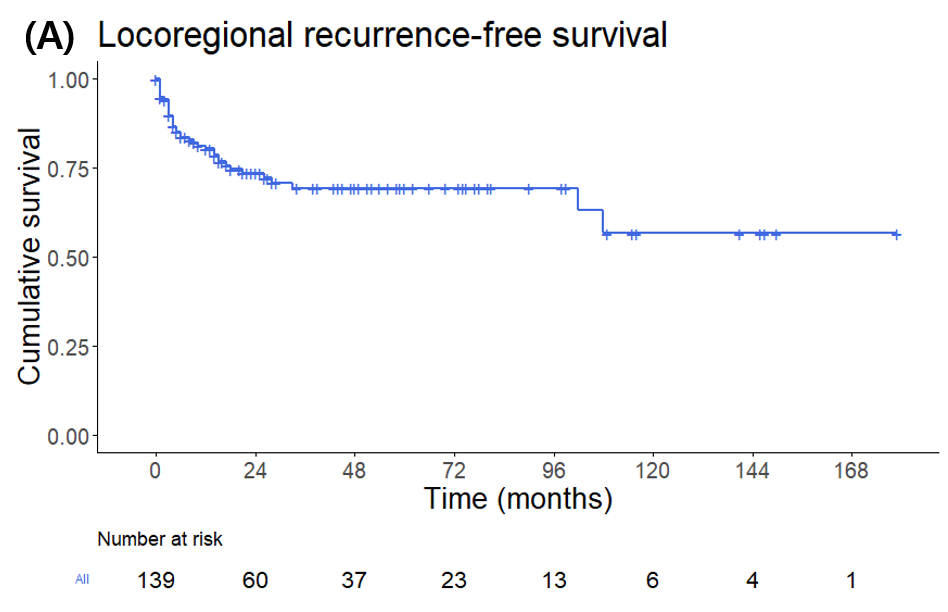


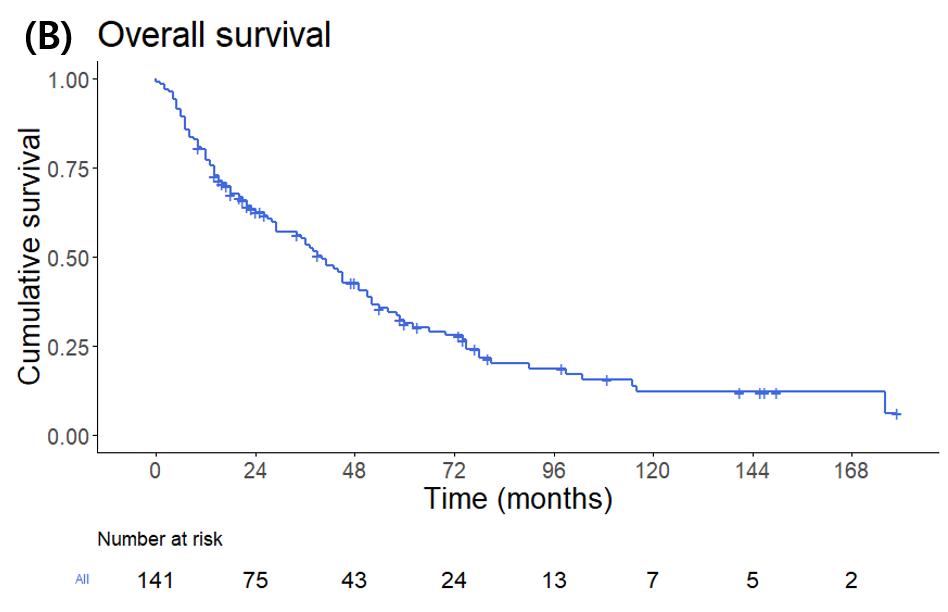

Supplement: Supplementary file 1 — Supplementary file1 (DOCX 168 KB) [file 464_2024_10691_MOESM1_ESM.docx]
